# B Vitamins in the nervous system: Current knowledge of the biochemical modes of action and synergies of thiamine, pyridoxine, and cobalamin

**DOI:** 10.1111/cns.13207

**Published:** 2019-09-06

**Authors:** Carlos Alberto Calderón‐Ospina, Mauricio Orlando Nava‐Mesa

**Affiliations:** ^1^ Center for Research in Genetics and Genomics (CIGGUR), GENIUROS Research Group, School of Medicine and Health Sciences Universidad del Rosario Bogotá Colombia; ^2^ Neuroscience Research Group (NEUROS), School of Medicine and Health Sciences Universidad del Rosario Bogotá Colombia

**Keywords:** B vitamins, biochemical action mechanism, neuropathy, pyridoxine, thiamine, vitamin B12

## Abstract

**Background:**

Neurotropic B vitamins play crucial roles as coenzymes and beyond in the nervous system. Particularly vitamin B1 (thiamine), B6 (pyridoxine), and B12 (cobalamin) contribute essentially to the maintenance of a healthy nervous system. Their importance is highlighted by many neurological diseases related to deficiencies in one or more of these vitamins, but they can improve certain neurological conditions even without a (proven) deficiency.

**Aim:**

This review focuses on the most important biochemical mechanisms, how they are linked with neurological functions and what deficits arise from malfunctioning of these pathways.

**Discussion:**

We discussed the main role of B Vitamins on several functions in the peripheral and central nervous system (PNS and CNS) including cellular energetic processes, antioxidative and neuroprotective effects, and both myelin and neurotransmitter synthesis. We also provide an overview of possible biochemical synergies between thiamine, pyridoxine, and cobalamin and discuss by which major roles each of them may contribute to the synergy and how these functions are inter‐related and complement each other.

**Conclusion:**

Taking into account the current knowledge on the neurotropic vitamins B1, B6, and B12, we conclude that a biochemical synergy becomes apparent in many different pathways in the nervous system, particularly in the PNS as exemplified by their combined use in the treatment of peripheral neuropathy.

## 1 BACKGROUND

The eight B vitamins B1 (thiamine), B2 (riboflavin), B3 (niacin), B5 (pantothenic acid), B6 (pyridoxine), B7 (biotin), B9 (folate), and B12 (cobalamin) form a group of chemically very heterogeneous essential substances, which have a wide variety of functions in the human body.^1^, ^2^, ^3^ Even though they are biochemically not related, referring to them as a group makes sense because they often naturally occur in the same foods^1^ and share the feature of being water‐soluble. Mammals are not able to synthesize B vitamins on their own; therefore, they must take them up in sufficient quantities with the diet. Even though most of them are produced by plants, they can be ingested indirectly via animal‐derived food like meat, dairy, and eggs. Only vitamin B12 is not produced by plants but by bacteria that colonize the foregut of ruminants or the colon of humans and thus can only be found in animal products like liver, fish, eggs, or dairy products. However, the vitamin B12 produced by bacteria in the colon of humans is not available for uptake because adsorption only takes place further up in the ileal mucosa through an intrinsic factor‐mediated mechanism.^2^, ^3^, ^4^ All B vitamins play crucial roles as coenzymes for enzymatic reactions in different biological systems.^1^, ^5^ Although those roles differ, they are closely inter‐related and complement each other.^2^, ^6^ In order to fulfill the coenzymatic function, the biologically active form of the respective vitamin (coenzyme) needs to bind to a corresponding protein (enzyme), thereby activating its enzyme function, so that the cellular processes can take place with the help of the newly formed holoenzyme complex.^2^, ^3^ Some of the B vitamins do not only contribute to important physiological functions in the whole human body but also possess neurospecific functions.^1^ These commonly called “neurotropic” B vitamins play special and essential roles both in the central nervous system (CNS) and the peripheral nervous system (PNS). It is well known that the diet and thus the supply of nutrients strongly affect normal functioning of CNS and PNS.^7^ In particular, vitamin B1, B6, and B12 are essential for maintaining the health of the nervous system.^2^, ^8^ Interaction between pyridoxine and cobalamin in the methionine cycle, as well as their participation in the citric acid cycle with other B vitamins, including thiamine, suggests that these three vitamins are linked from a biochemical point of view.^2^, ^9^ Indeed, a significant association between cognitive impairment and methionine‐homocysteine cycle dysfunction indicated by low levels of vitamins B6 and B12 has been found.^9^, ^10^, ^11^ Evidence suggests that a significant proportion of the population suffers from deficiencies and insufficiencies of one or more of these neurotropic B vitamins. The importance of B vitamins in the context of nerve function is highlighted by the numerous neurological diseases, such as Wernicke's encephalopathy, depression, beriberi, seizures, subacute combined degeneration of the spinal cord, or peripheral neuropathy (PN), that are related to a deficiency in one or more of these neurotropic B vitamins.^2^, ^6^, ^8^, ^9^, ^12^, ^13^ However, the significance of these vitamins is also emphasized by the fact that they can improve certain neurological conditions even if no (definite) deficiency can be proven.^2^, ^14^, ^15^ Indeed, several reports indicate that the specific supplementation with the combination of vitamins B1, B6, and B12 interacts synergistically to improve neuropathy, motor control, nociceptive, and neuropathic pain.^16^, ^17^, ^18^, ^19^ The present review aims to compile the most important biochemical pathways of the B vitamins, focusing on thiamine, pyridoxine, and cobalamin, and link them with neurological functions and symptoms related to deficiencies. We also provide an overview of possible biochemical synergies between these neurotropic vitamins and discuss major roles by which they may contribute to this synergy.

## 2 BIOCHEMICAL MODE OF ACTION AND ROLE IN THE NERVOUS SYSTEM

### 2.1 Vitamin B1 (thiamine)

Vitamin B1, also known as thiamine, has long been known to be associated with functions in the nervous system. The connections between thiamine deficiency and the development of fatal conditions such as beriberi, a syndrome compromising the PNS by polyneuritis and/or cardiovascular symptoms, and the neuropsychiatric Wernicke‐Korsakoff syndrome, characterized by encephalopathy and psychosis, were already recognized in the early to mid‐20th century.^3^, ^20^

In general, thiamine is essential for many physiological functions and is, among other roles, involved in glucose metabolism, the maintenance of nerve membrane function, and the synthesis of myelin and several types of neurotransmitters (eg, acetylcholine, serotonin, and amino acids).^20^, ^21^, ^22^, ^23^

However, the most important function of thiamine is considered to be that it largely contributes to the cellular energy metabolism and, as an essential cofactor in the conversion of carbohydrates, helps providing energy to nerve cells.^24^, ^25^ This constant supply of energy is essential because nerve cells, especially in the brain, consume a great amount of energy to maintain their functions and, for example, prevent premature aging, but can hardly store high‐energy compounds themselves.^7^ To be more precise, one of the main activities of thiamine is to enable biochemical steps in the energy‐creating processes pentose phosphate pathway, glycolysis, and Krebs cycle (citric acid cycle). These processes supply the nerves with energy mainly in the form of adenosine triphosphate (ATP) or nicotinamide adenine dinucleotide phosphate (NADPH), which in turn are essential for numerous other cellular processes and reactions in nerves.^20^, ^23^, ^26^ By means of that, vitamin B1 is also indirectly needed for the energy‐consuming synthesis of nucleic acids, neurotransmitters, and myelin.^20^, ^23^, ^24^, ^25^, ^26^ Therefore, thiamine even contributes to nerve conduction velocity because it participates in the maintenance of myelin sheaths.^23^, ^25^ Because the mentioned pathways not only produce energy but also provide reducing power, thiamine is thought to also have an antioxidative—thereby protective—effect on nerve cells.^3^, ^20^

In addition to its coenzymatic functions, thiamine is also believed to be directly involved in nerve stimulation in a non‐coenzymatic way due to its interference with the structure and function of cellular membranes and its ability to regulate ion channels.^22^, ^23^, ^25^, ^27^ Furthermore, through its antioxidative properties, sufficient amounts of thiamine may even prevent cell damage resulting from hyperglycemia.^25^, ^28^

**Figure 1:**
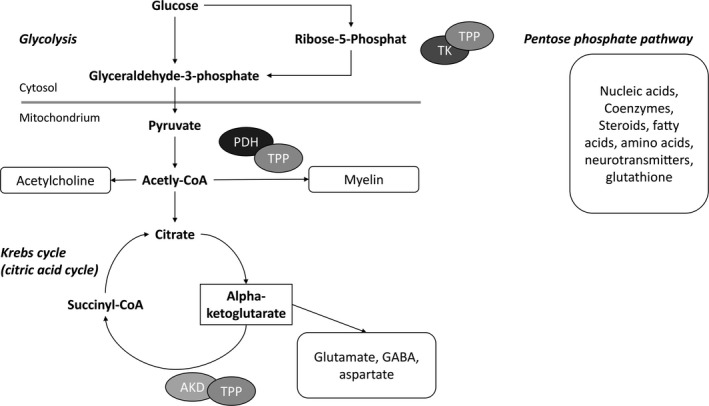
Biochemical mechanism of action of vitamin B1 (thiamine). Modified and simplified illustration based on.^24^, ^26^ TPP, thiamine pyrophosphate; TK, transketolase; PDH, pyruvate dehydrogenase; AKD, alpha‐ketoglutarate dehydrogenase; CoA, coenzyme A; GABA, gamma‐aminobutyric acid

At the molecular level, after being taken up by the cells by a usually active process, free thiamine is initially phosphorylated to form biochemically active thiamine diphosphate (TDP), synonymously known as thiamine pyrophosphate (TPP). TPP acts as a coenzyme for thiamine‐using enzymes in three major pathways of glucose metabolism; that is, for transketolase (TK) in the pentose phosphate pathway, for pyruvate dehydrogenase (PDH) in the glycolysis, and for alpha‐ketoglutarate dehydrogenase (AKD) in the Krebs cycle^24^, ^25^, ^26^ (Figure 1). Each of these enzymes can only fulfill its purpose as a holoenzyme made up of several constituents. Therefore, the addition of thiamine to the complex is crucial for the enzymes' functionality. The pentose phosphate pathway, which generates the sugar molecule ribose‐5‐phosphate and the energy source NADPH, uses the TPP‐activated TK in the cytosol to convert ribose‐5‐phosphate to glycerinaldehyde‐3‐phosphate. The substrates of the pentose phosphate pathway are then used for the synthesis of nucleic acids, complex sugar molecules, coenzymes, steroids, fatty acids, amino acids, neurotransmitters, and glutathione. Through its operation, TK also connects the pentose pathway with the glycolysis.^24^, ^26^, ^29^

In contrast, the TPP‐activated enzymes PDH and AKD hold special functions in glycolysis and the Krebs cycle, which in particular provide ATP for cell energy. Further, PDH induces the formation of acetyl coenzyme A (CoA), a precursor of the neurotransmitter acetylcholine, and helps producing myelin that is needed to enwrap the axons of nerve cells.^3^, ^26^ AKD in the Krebs cycle, on the other hand, helps maintaining the levels of neurotransmitters (ie, glutamate, GABA, and aspartate) and also supports protein synthesis.^26^

In the case of a vitamin B1 deficiency, activity levels of all three enzymes mentioned above are biochemically impaired; however, TK activity may be most sensitive and AKD activity one of the earliest changes. As vitamin B1 is essential for the production of energy (ATP and NADPH) and a normal function of nerve cells, its deficiency can cause neurons to die or become damaged.^30^ Thiamine deficiency affects both the CNS and the PNS and can manifest clinically in multifaceted ways. In general, neurological symptoms of thiamine deficiency include confusion, psychomotor retardation, lack of insight, impaired retentive memory and cognitive function, confabulation, ataxia, and the loss of vibration and position sense.^21^, ^24^ If thiamine is not present in sufficient quantity for the CNS, sensitive areas of the brain such as the thalamus and the mamillary bodies (part of the hypothalamus) suffer damage.^31^ Wernicke's encephalopathy and Korsakoff's psychosis (often referred to as Wernicke‐Korsakoff syndrome) can certainly be considered the most serious CNS manifestations of thiamine deficiency.^20^, ^30^ In Wernicke's encephalopathy, for instance, thiamine deficiency is thought to trigger apoptotic cell death due to N‐methyl‐D‐aspartate (NMDA) toxicity and thereby induce neurological symptoms.^32^ In the PNS, typical manifestations of thiamine deficiency include polyneuritis and paralysis, as occurs in dry beriberi.^3^, ^20^ In the sensory system, it influences the tactile sensation, causes pain, changes the temperature sensitivity, and leads to the loss of vibratory sense. In the motor system, paralysis typically begins in the tips of the lower extremities and spreads progressively. It involves increased muscle weakness, affected tendon reflexes, and atrophy of the leg muscles.^21^ Thiamine deficiency nowadays hardly affects the general population in developed countries, but certain vulnerable populations are very often deficient or show suboptimal levels.^22^, ^24^ For example, it is assumed that this applies to up to 80% of alcoholics,^23^ up to 98% of diabetics,^33^ and around one‐third of dialysis patients with altered mental status.^34^

Because vitamin B1 is largely involved in pathways that also create reducing power in cells, deficiency will cause cells to be exposed to oxidative stress, which can lead to cell damage and cell death and contribute to further symptoms and comorbidities.^20^, ^24^, ^25^, ^26^

In summary, these examples clearly show how important thiamine is for the nervous system function due to its activating role for neuronal excitability and metabolism as well as antioxidative effects.

### 2.2 Vitamin B6 (pyridoxine)

Vitamin B6 (pyridoxine) has been discovered in 1934 and has so far been associated with over 140 coenzymatic functions.^3^, ^31^, ^35^ Although its role goes far beyond, it is particularly well known for its important function in the synthesis of neurotransmitters like dopamine from L‐DOPA, serotonin from 5‐HTP, and gamma‐aminobutyric acid (GABA) from glutamate.^1^, ^3^, ^5^, ^31^, ^36^, ^37^ According to its function for the previously mentioned neurotransmitters (and others), pyridoxine affects the adrenergic, the serotonergic, and the glutamatergic system. Pyridoxine can also be attributed a neuroprotective role which appears to be mainly linked with its ability to regulate the glutamatergic system and thus GABA and glutamate levels. Since GABA serves as the major inhibitory neurotransmitter, it seems obvious that GABA deficiency can lead to serious consequences, such as seizures. Increased levels of the GABA precursor glutamate, an excitatory neurotransmitter, can be linked with seizures, whereas the application of GABA or pyridoxine can end seizure activity.^1^, ^3^, ^36^ In addition, pyridoxine administration even attenuates the excitotoxicity of the neurotoxin domoic acid.^38^ Beyond that, it has been shown that vitamin B6 is essential during gestation and postnatal brain development, probably also through the regulation of GABA levels. Rats exposed to vitamin B6 deficiency during this time showed significantly lower GABA levels and permanently damaged brains.^39^

While only nonphosphorylated B6 vitamers can cross cell membranes, including the blood‐brain barrier,^40^ and can therefore be taken up by cells, vitamin B6 is intracellularly phosphorylated to form the active interconvertible 5′‐phosphate esters pyridoxine 5′‐phosphate (PNP), pyridoxal 5′‐phosphate (PLP; most important coenzyme variant), and pyridoxamine 5′‐phosphate (PMP).^36^ Beyond its essential role in neurotransmitter production, PLP also acts as a coenzyme in one‐carbon unit generation and homocysteine metabolism, supports carbohydrate and fat synthesis as well as breakdown, and helps releasing food‐bound energy that is needed for the metabolism of proteins and amino acids.^3^, ^31^, ^36^, ^41^ Besides, PLP also serves as a cofactor in sphingolipid synthesis and is thereby important for myelin formation.^5^, ^36^, ^38^

**Figure 2:**
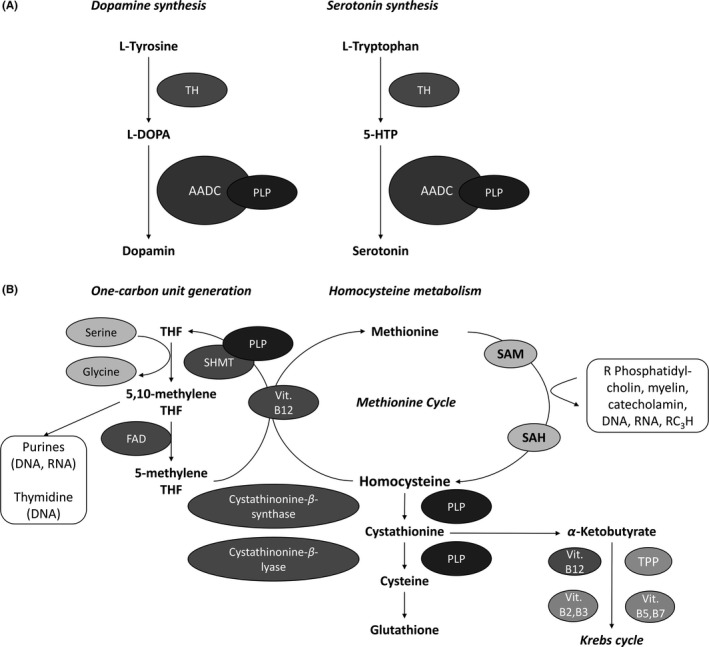
Biochemical mechanism of action of vitamin B6 (pyridoxine). A, Role of PLP on Dopamine and Serotonin Synthesis. B, Role of PLP and Vit. B12 on one‐carbon unit metabolism and Hcy metabolism. Role of B Vitamins in the interlinked methionine and citric acid cycles. Modified and simplified illustration based on.^36^, ^42^, ^43^ TH, tyrosine hydroxylase; AADC, aromatic L‐amino acid decarboxylase; PLP, pyridoxal 5′‐phosphate; 5‐HTP, 5‐hydroxytryptophan; THF, tetrahydrofolate; SHMT, serine‐hydroxymethyltransferase; FAD, flavin adenine dinucleotide; SAM, S‐adenosylmethionine; SAH, S‐adenosylhomocysteine; R, methyl group acceptor

With regard to neurotransmitter synthesis, PLP helps, for instance, catalyzing the final production step of dopamine and serotonin, that is, the enzymatic decarboxylation of L‐DOPA to dopamine and of 5‐HTP to serotonin (Figure 2A). In both pathways, successful formation of the neurotransmitters depends on the action of aromatic L‐amino acid decarboxylase (AADC), which in turn essentially depends on PLP.^37^, ^42^, ^43^

In the one‐carbon unit metabolism, PLP‐activated serine‐hydroxymethyltransferase (SHMT) catalyzes the process in which one‐carbon units are generated from serine and activated through association with tetrahydrofolate (THF). This pathway forms 5,10‐methylene‐THF for nucleic acid synthesis and the methyl donor 5‐methyl‐THF, which is needed for protein synthesis and to methylate homocysteine (Hcy) to methionine in a process that also depends on vitamin B12 and folate. A great proportion of the formed methionine is converted to S‐adenosylmethionine (SAM), a universal donor of methyl groups needed for the synthesis of DNA, RNA, hormones, neurotransmitters, membrane lipids, proteins, and others. Being an intermediate compound of the methionine metabolism, Hcy can be disposed by two pathways. When methionine is in excess or cysteine is required, it will be disposed via the intermediates cystathionine and cysteine to glutathione. In methionine deficiency, on the other hand, it is remethylated to methionine in the above‐described way^36^, ^44^ (Figure 2B).

The role of pyridoxine in the nervous system is clearly demonstrated by its use in the treatment of pyridoxine dependency seizures—an inborn abnormality in infants with seizures not responding to common anticonvulsants.^3^, ^36^ Due to its important function as a coenzyme in pathways responsible for the synthesis of neurotransmitters and myelin, vitamin B6 deficiency can severely impair the CNS and the PNS.^1^, ^3^, ^5^, ^36^ Biochemically, in a partial deficiency of vitamin B6, some enzymes may be more affected than others, resulting in greater depletion of some neurotransmitters and thereby imbalances between the levels of different neurotransmitters.^38^ Neurological symptoms of deficiency generally range from impaired cognitive function, convulsive seizures, depression, and even premature aging of neurons (CNS effects) to carpal tunnel syndrome and PN with symptoms like paresthesia, burning and painful dysesthesias, and thermal sensations (PNS effects).^1^, ^3^, ^5^, ^7^, ^35^, ^36^ Treating these conditions with pyridoxine is clearly useful, even though the intake of extremely high doses over long periods can itself trigger sensory neuropathy.^3^, ^36^ However, even in these circumstances, symptoms resolve after withdrawal and no permanent damage to the nervous system has so far been described.^3^ Like thiamine deficiency, vitamin B6 deficiency is also rare in the healthy general population in countries with high nutritional standards but frequently affects hemodialysis patients (over 80%),^45^ particularly if they are uremic. In addition, increased amounts of vitamin B6 are needed during pregnancy to ensure fetal brain development,^46^ and pyridoxine supplementation may even reduce nausea during early pregnancy.^47^

In summary, pyridoxine strongly contributes to the proper functioning of the nervous system by facilitating neurotransmitter and myelin synthesis, and also controlling glutamate excitability and neuronal metabolism.

### 2.3 Vitamin B12 (cobalamin)

The discovery of vitamin B12 (cobalamin) can be attributed to a disease that already attracted attention long time ago and became known as pernicious anemia.^3^, ^48^, ^49^ Even though it first became famous for its role in hematopoiesis, cobalamin also plays an essential role as a coenzyme in many biochemical processes that maintain or restore the health of the nervous system. Thus, vitamin B12 is especially awarded a function in the DNA synthesis of myelin‐producing oligodendrocytes and the synthesis of myelin.^48^, ^49^, ^50^, ^51^ The myelin sheath surrounds the axons of many nerves and serves as an electrical insulation, thereby facilitating fast conduction velocity. Through this important contribution to myelin formation and remyelination, it significantly supports the regeneration of nerves after an injury.^8^, ^50^ In addition to this major role, cobalamin is involved in Hcy metabolism, nerve metabolism (transmethylation processes), fatty acid and nucleic acid synthesis, energy production as well as cell maturation processes and even supports the maintenance of an intact gastrointestinal mucosa.^48^, ^49^, ^50^, ^51^, ^52^, ^53^ Since the level of cobalamin also affects the amount of reduced glutathione with antioxidant functions in the erythrocytes and in the liver, the lower availability of reduced glutathione in cobalamin deficiency may expose cells to increased oxidative stress.^7^

**Figure 3:**
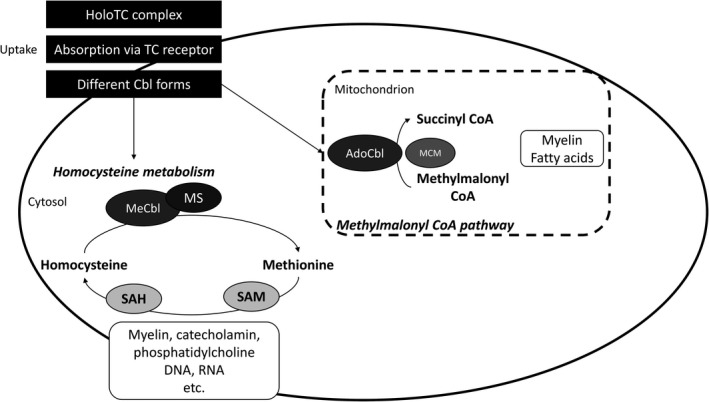
Biochemical mechanism of action of vitamin B12 (cobalamin). Modified and simplified illustration based on.^48^, ^54^ HoloTC, holotranscobalamin; TC, transcobalamin; Cbl, cobalamin; MeCbl, methylcobalamin; MS, methionine synthase; SAH, S‐adenosylhomocysteine; SAM, S‐adenosylmethionine; AdoCbl, adenosylcobalamine; MCM, methylmalonyl CoA mutase; CoA, coenzyme A

The pathway from nutritional vitamin B12 intake to cellular usability of the coenzyme forms is complex and involves several steps during which cobalamin (Cbl) is bound and transported through the intestine and the blood by different proteins such as haptocorrin, intrinsic factor, and transcobalamin II. The holotranscobalamin complex is finally absorbed by the target cell after binding to the transcobalamin receptor.^48^, ^54^ Cbl naturally occurs in several forms differing only in their prosthetic groups, all of which are cleaved and metabolized to the coenzyme variants methylcobalamin (MeCbl) and adenosylcobalamin (AdoCbl) after uptake.^49^, ^51^, ^53^, ^55^ It is important to understand that all of these forms first have to be converted to the Cbl core structure before they are later newly assembled into the active coenzymes in the body. Therefore, direct intake of the coenzyme forms does not seem to be associated with advantages.^55^ Cbl forms in the mitochondria are changed in a complex enzymatical process into AdoCbl, which supports the enzyme methylmalonyl CoA mutase (MCM) and thereby helps catalyzing the formation of succinyl CoA—an important intermediate of the Krebs cycle—from methylmalonyl CoA (Figure 3). Methylmalonyl CoA emerges when odd‐chain fatty acids, cholesterol, and ketogenic amino acids are metabolized.^3^, ^54^, ^56^ In contrast to the processes in the mitochondria, the equally complex enzymatical conversion of Cbl to MeCbl only occurs in the cytosol. Here, the enzyme methionine synthase (MS) requires MeCbl as a cofactor to methylate the amino acid Hcy to methionine (Figure 3), which is needed to sustain adequate synthesis of proteins, DNA, and neurotransmitters.^3^, ^48^, ^51^, ^53^, ^54^, ^55^ If Cbl is deficient in the cell, plasma concentrations of methylmalonic acid—a functional marker of vitamin B12 deficiency—and Hcy will rise. Moreover, deficiency also leads, among other things, to defect in myelin synthesis and the incorporation of abnormal fatty acids into neuronal.^48^, ^49^, ^51^, ^53^, ^54^

Because vitamin B12 is involved in so many essential pathways, its deficiency is a tremendous health problem. However, symptoms strongly differ in severity and can manifest as mild conditions or life‐threatening disorders.^57^ Neurological deficiency disorders include but are not limited to subacute combined sclerosis of the spinal cord, polyneuritis, neuropathy, myelopathy, optic nerve atrophy, and impaired cognitive function and are mainly related to impaired neurotransmitter production, myelin lesions, or increased Hcy and methylmalonic acid levels.^3^, ^49^, ^51^, ^54^, ^57^, ^58^, ^59^, ^60^ Neuronal demyelination is thought to be mainly caused when the universal methyl donor SAM is less available. SAM synthesis critically depends on vitamin B12 and has various important functions in the nervous system, including myelin as well as neurotransmitter synthesis. Demyelination generally affects both peripheral and central nerves but especially the long tracts of white matter in the posterior and lateral columns of the spinal cord, which contain sensory fibers for the vibration and position sense. However, motor fibers can also become demyelinated.^3^ Affected persons may suffer from symptoms such as symmetric dysesthesia, disturbance of position sense, spastic paraparesis or tetraparesis, paresthesias, numbness in limbs, and difficulties in activities of daily living like writing or buttoning.^51^, ^53^, ^54^, ^57^ Vitamin B12 deficiency appears to be particularly common in the elderly with estimates as high as 30‐40% and may often be due to malabsorption. In addition, vegetarians and particularly vegans often show suboptimal vitamin B12 levels but do not necessarily develop a clinical deficiency.^3^, ^49^, ^51^, ^57^

Overall, it can also be summarized for vitamin B12 that it is essential for the nervous system, particularly with regard to myelin synthesis, nerve metabolism, and neuronal regeneration.

## 3 THE ROLE OF NEUROTROPIC B VITAMINS IN DIFFERENT REGIONS OF THE NERVOUS SYSTEM

As outlined in this review, neurotropic B vitamins play important roles both in the CNS and the PNS. While the biochemical mechanisms at the cellular level are identical in both systems, the phenotypic manifestations of deficiencies differ.^1^, ^3^

In the CNS (ie, the brain and the spinal cord), one of the most prominent roles of neurotropic B vitamins (particularly vitamins B6 and B12 as well as the herein not described B9) derives from their contribution to the folate and Hcy metabolism. Deficiencies in these vitamins are associated with increased Hcy levels, which are assumed to have neurotoxic effects. By promoting oxidative stress and neurodegeneration, increased Hcy may be a risk factor for dementia, cognitive decline, and Alzheimer's disease.^2^, ^9^, ^12^, ^53^ In addition, dietary supplementation with B Vitamins may have beneficial effects in other neurological conditions such as anxiety, stress‐related disorders, and multiple sclerosis.^61^, ^62^

Also in the PNS, neurotropic B vitamins contribute to the maintenance of optimal nerve functioning. Deficiencies can result in the development of disorders of the peripheral nerves, for example, peripheral neuropathies. Evidence suggests that these vitamins also play a role in the regeneration of injured nerves, as shown in several animal studies (for examples see^63^, ^64^). In addition, studies in humans have shown that treatment with neurotropic B vitamins effectively relieved symptoms of neuropathy in different patient groups (for examples see^19^, ^64^). Patients with such conditions may even benefit from pharmacological B vitamin doses if no clear diagnosis of deficiency can be established or only suboptimal B vitamin levels (“marginal deficiency”) are detected.^2^, ^14^, ^15^ This assumption is supported by a recent prospective, non‐interventional study of Hakim et al, in which patients with PN of different etiology were treated with high‐dose B vitamins (B1, B6, and B12) for a period of 90 days without prior determination of B vitamin levels; all groups benefited significantly from the treatment and felt progressive relief of different symptoms such as pain, burning, paresthesia, and numbness.^19^ However, the benefit of neurotropic B vitamins in patients with neuropathy should in the future also be confirmed by randomized controlled trials.

## 4 **SYNERGISTIC EFFECT OF THE COMBINATION OF NEUROTROPIC B VITAMINS B1**, **B6, AND B12 WITH EMPHASIS ON THE PNS**

It needs to be stressed that vitamin B1, B6, and B12 most likely hold synergistic biochemical roles in the nervous system, that is, neither of them can replace one of the others. Table 1 provides an overview on the major implications in overlapping biochemical pathways important for the nervous system, pointing to a synergistic effect as a logical consequence of these overlaps. Considering the fact that PN of different etiologies is believed to be a multifactorial process involving different factors like oxidative stress and demyelination,^65^, ^66^, ^67^, ^68^, ^69^ the hypothesis of synergy becomes even more likely. We postulate that the synergistic function of neurotropic B vitamins in the PNS may be primarily due to prominent functions of each vitamin. While we assume that vitamin B1 is mainly needed as an antioxidant in this context, vitamin B6 may be primarily involved in a neuroprotective and vitamin B12 in a myelin‐regenerating role. However, the idea of synergistic effects between B vitamins has already been discussed by other authors.^6^, ^8^ Nevertheless, clinical studies that support the hypothesis are needed and should directly compare the combination of the neurotropic B vitamins B1, B6, and B12 with the individual vitamins in humans suffering from PN. In contrast, results from animal studies suggest the correctness of the hypothesis. Thus, evidence for the practical synergistic action in the PNS was impressively demonstrated by Jolivalt et al, who showed that none of the individual B vitamins (B1, B6, and B12) was as effective in alleviating neuropathic pain and restoring nerve function in rats with experimentally induced diabetic neuropathy as the combination of the three when comparing high‐dose administration.^70^

**Table 1:** Overview on major biochemical mechanisms of action of vitamins B1, B6, and B12 for nerve function

Vitamin	Processes	Coenzyme for	Implication in nervous system
B1 (thiamine)	Glycolysis Pentose phosphate pathway Krebs cycle (citric acid cycle)	Pyruvate dehydrogenase Transketolase Alpha‐ketoglutarate dehydrogenase	Provide energy to nerve cells which are needed for synthesis of nucleic acids, neurotransmitters, and myelin
B6 (pyridoxine)	One‐carbon unit metabolism Hcy metabolism Dopamine and serotonin synthesis	Serine‐hydroxymethyltransferase Cystathionine‐beta‐synthase/lyase Aromatic L‐amino acid decarboxylase	Metabolism of amino acids, neurotransmitters, and DNA/RNA
B12 (cobalamin)	Hcy metabolism Methymalonyl CoA pathway	Methionine synthase Methylmalonyl CoA mutase	Metabolism of fatty acids, amino acids, neurotransmitters, myelin, and DNA/RNA

Note

Overview not exhaustive. Content based on references.^3^, ^6^, ^23^, ^26^, ^36^, ^49^, ^54^, ^57^

## 5 FINAL REMARKS AND CONCLUSION

As highlighted here, the neurotropic vitamins B1, B6, and B12 have different neurospecific functions in the nervous system. They are all important for the maintenance of normal neurological functions due to different biochemical modes of action, especially as coenzymes but also beyond,^1^, ^3^ and can effectively be used in combination for the treatment of PN in humans.^19^, ^64^ However, the exact mechanisms of action of these B vitamins in PN are still not clarified in detail and require further research.

In summary, vitamin B1 is particularly needed as a cofactor in glucose metabolism and thereby indirectly supports the synthesis of nucleic acids, neurotransmitters, myelin, etc by providing energy for these processes. In addition, it is assumed to contribute to antioxidative mechanisms.^24^, ^26^ Vitamin B6, most importantly, functions as a coenzyme in the synthesis of neurotransmitters needed for synaptic transmission (eg, dopamine, serotonin, GABA) and holds a neuroprotective role based on its importance for the glutamatergic system.^3^, ^5^, ^36^ With regard to neuropathy, the main role of vitamin B12 is attributed to the synthesis of myelin, which allows for the regeneration of peripheral nerves.^48^, ^49^, ^50^, ^51^

Taking into account the current knowledge on the neurotropic vitamins B1, B6, and B12, we conclude that they form a biochemical synergy in many different pathways in the nervous system, particularly in the PNS as exemplified by their combined use in the treatment of PN. It is important to start considering B vitamins in future clinical studies as a therapeutic and neuroprotective approach for both peripheral neuropathies and several brain disorders.

## CONFLICT OF INTEREST

Carlos Calderon‐Ospina holds consultative activities for Merck Selbstmedikation GmbH, an affiliate of the P&G Group of Companies.
